# Nonstructural Protein 1 Mediates HMGB1 Release by Targeting Histone H1.0 After Respiratory Syncytial Virus Infection In Vivo and In Vitro

**DOI:** 10.1007/s10753-025-02285-6

**Published:** 2025-05-14

**Authors:** Na Zhou, Siyi Che, Hui Zhai, Xiaohong Xie, Enmei Liu, Jun Xie

**Affiliations:** 1 Department of Respiratory Medicine, Children’S Hospital of Chongqing Medical University; National Clinical Research Center for Child Health and Disorders; Ministry of Education Key Laboratory of Child Development and Disorders; China International Science and Technology Cooperation Base of Child Development and Critical Disorders; Chongqing Key Laboratory of Child Rare Diseases in Infection and Immunity, Chongqing, 400014 China; 2 Department of Pediactirc, Bishan Hospital of Chongqing Medical University, Bishan County of Chongqing, Chongqing, 402760 China

**Keywords:** RSV, HMGB1, Nonstructural protein 1, Histone H1.0

## Abstract

High mobility group box-1 (HMGB1) is implicated in airway inflammation during the late phase of respiratory syncytial virus (RSV) infection. Despite its recognized role, the specific mechanism underlying its release post-RSV infection remains ambiguous. The nonstructural protein 1 (NS1) has been associated with interactions with numerous host proteins, affecting diverse physiological processes, and it is speculated to be involved in the release of HMGB1. We utilized an in vivo model of RSV-infected mice and an in vitro model of RSV-infected A549 and 16HBE cells to investigate the role of NS1 in promoting HMGB1 release. Small interfering RNA was employed to deplete NS1, while lentiviral vectors were used for NS1 overexpression. The interaction between NS1 and H1.0 was confirmed by immunofluorescence analysis, immunoprecipitation, GST pull-down assays, surface plasmon resonance analysis and in silico study. Our study revealed that silencing the NS1 gene reduced the levels of HMGB1 protein and suppressed airway inflammation during the late stage of RSV infection. Depletion of NS1 led to decreased levels of intracellular and extracellular HMGB1 in A549 and 16HBE cells, while over-expression of NS1 increased HMGB1 expression. Furthermore, NS1 and HMGB1 directly interacted with histone H1.0, as confirmed by GST pull-down, surface plasmon resonance and in silico analyses. Overexpression of NS1 disrupted the binding of HMGB1 to H1.0, while silencing of NS1 enhanced their interaction. The research findings indicate that NS1 interacts with H1.0, thereby inhibiting the binding of HMGB1 to H1.0. Consequently, this interaction results in the release of HMGB1 into both the cytoplasm and the extracellular space.

**Supplementary Information:**

The online version contains supplementary material available at 10.1007/s10753-025-02285-6.

## Introduction

Respiratory syncytial virus (RSV) is the predominant viral pathogen responsible for acute lower respiratory tract infections in children under the age of 5 worldwide. The global annual hospitalization rate due to RSV is approximately 9.7%, leading to the deaths of nearly 60,000 children [1]. Beyfortus and Palivizumab are antiviral medications against RSV currently in use. As an RNA virus with a single-stranded negative-sense genome that encodes 11 proteins, RSV expresses nonstructural protein 1 (NS1) primarily in the early stages of infection. NS1 interferes with both IFN production and signaling by affecting the type I interferon receptor or inhibiting the mitochondrial antiviral signaling protein (MAVS) pathway [2, 3]. Despite its role in enhancing viral replication, NS1 is not involved in viral packaging and is absent in mature virus particles, making it an infrequent target for subunit vaccines. Silencing the NS1 gene using plasmids early in infection has been shown to reduce RSV replication, alleviate lung inflammation, and decrease airway hyper-responsiveness (AHR) in mouse models [4]. NS1 interacts with various host proteins, including Mediator subunits and RNApol II, influencing multiple physiological processes [5]. Our previous study revealed that NS1 competitively binds to the host transporter IPO13, impairing the nuclear translocation of the glucocorticoid receptor, resulting in reduced sensitivity to glucocorticoid therapy post RSV infection [6]. Therefore, further investigation is warranted to understand the significant role NS1 plays in the pathophysiological processes following RSV infection.

HMGB1 is a conserved non-histone DNA-binding protein found in the nucleus, cytoplasm, and extracellular space. In the nucleus, HMGB1 serves as a chromatin structural factor, contributing to chromosome stability, nucleosome stabilization, and gene transcription regulation [7, 8]. Outside the cell, HMGB1 can trigger the release of inflammatory mediators such as TNF-α, IL-1, and IL-8 [7, 9]. Studies by our research team have shown that HMGB1 levels rise in the later stages specific to RSV infection in mice, even in the absence of active viral replication. Blocking HMGB1 with monoclonal antibodies markedly decreases cytokine production, airway inflammation, and airway hyper-responsiveness [10]. The extracellular release of HMGB1 can occur passively during necrotic cell death or actively by macrophages in response to infections, inflammation, or oxidative stress. Adenovirus protein VII has been reported to interact with both chromosomes and HMGB1, increased the binding of HMGB1 to chromatin and hindering its extracellular release [11]. Moreover, research by Chen et al. [12] demonstrated that the X protein of hepatitis B virus (HBx) induces the translocation and release of HMGB1 from the nucleus to the cytoplasm and extracellular environment, with HMGB1 levels correlating with viral DNA replication. In vitro experiments have shown that NS1 interacts directly with histone H2BD, leading to histone ubiquitination and subsequent upregulation of HOX gene expression after RSV infection [13]. However, the impact of NS1 on HMGB1 expression and release remains unclear.

This study aimed to investigate the association between NS1 and HMGB1 release in RSV-infected A549 cells. Both an in vivo model of RSV-infected mice and an in vitro model of RSV-infected A549 cells were utilized to determine the impact of NS1 on HMGB1 expression and release. The results showed that NS1 induces HMGB1 expression and directly interacts with histone H1.0, thereby inhibiting HMGB1 binding to H1.0 and favoring its subsequent release. These findings highlight the pivotal role of NS1 in HMGB1 expression and release from airway epithelial cells, underscoring its significance in participating airway inflammation following RSV infection.

## Methods and Materials

### Mice

Female BALB/c mice (6–8 weeks old) were obtained from the Chongqing Medical University Animal Laboratory and housed as previously described [6]. All animal experimental protocols received approval from the Ethics Committee at the Children’s Hospital of Chongqing Medical University(Approval number:20240412003), and the animal studies were conducted in compliance with the Chinese Council on Animal Care.

### Cell Culture and Virus Infection

The A549 and 16HBE cell lines(ATCC,USA) were used for the RSV infection experiments. The cells were cultured in Dulbecco’s Modified Eagle Medium (DMEM) supplemented with 10% fetal bovine serum. For viral adsorption, A549 or 16HBE cells A549 or 16HBE cells were cultured overnight in 12- or 6-well plates and subsequently infected with RSV for two hours, using a multiplicity of infection (MOI) of 1. The medium was removed and replaced with fresh medium containing 10% serum. The infected cells was then cultrued for another 24 or 36 h at 37 °C.

### Mouse RSV Infection

Mice were inoculated with RSV as described previously [6]. We utilized the A2 strain of human RSV (VR-1540, American Type Culture Collection, USA) without lipopolysaccharide (LPS), with intranasal inoculation with 1.5 × 10^7^ plaque forming units (PFU) of RSV in a 100-μL volume or sham infection with 100 μL of HEp-2 suspension without virus.

### Bronchoalveolar Lavage Fluid (BALF) Inflamatory Cell Count

The BALF was collected to evaluate inflammatory cell as described in our previous works [6, 10]. Briefly, mice were anesthetized on day 21 after RSV infection, and the lungs were lavaged, in situ, six times with 0.5 ml of ice-cold PBS. The BAL fluid was centrifuged (2500 rpm, 5 min). The supernatants were stored at −80℃. The cellular pellets were resuspended in 1 ml of PBS, and the total cell number was counted. For differential cell counts, 250 μl of the resuspended cells was spun onto microscope slides, airdried, and stained. For each sample, 200 cells were counted for the number of macrophages, neutrophils, lymphocytes and eosinophils.

### Cytokine Measurement

The concentration of HMGB1 in the cultured cell supernatants was determined by using the human HMGB1 Enzyme-Linked Immunosorbent Assay (ELISA) Kit (Shino-Test, Kanagawa, Japan) according to the ELISA kit instructions.

### Lung Histopathology

Mice were euthanized by cervical dislocation. Their left lung tissue were fixed in 10% (vol/vol) neutral buffered formalin for 24 h and then embedded in paraffin, cut into 5-μm thickness, and stained with hematoxylin and eosin (H&E). The degree of lung tissue damage was evaluated by assigning a value of 0 for no inflammation, 1 for mild inflammation, 2 for moderate inflammation, and 3 for severe inflammation.

### Knockdown Experiments

RSV NS1 small interfering RNA (siRNA) and negative control siRNA were purchased from GeneChem (Shanghai, China) as used previously [6]. The siRNA sequences were: siNS1: 5´-GGCAGCAATTCATTGAGTATG-3´; siCON (negative control): 5´-TTCTCCGAACGTGTCACGT-3´. siNS1 or siCON was transfected cells by using Lipofectamine 3000 Reagent (Invitrogen, USA). The cells were incubated with RSV for 6 h, after which the plasmid (siNS1 or siCON) was transfected for another 24 h. Mice were inoculated intranasally with the siNS1 or siCON plasmids using Entranster In Vivo Transfection Reagent (Engreen Biosystem, New Zealand) in accordance with the manufacturer’s recommendations one day before RSV infection and two days after RSV infection, respectively.

### Lentiviral Construct Assay

The NS1 lentivirus (LV5-NS1-GFP) and negative control (LV5-CON-GFP) encoding enhanced green fluorescent protein were purchased from GenePharma (Shanghai, China). A549 cells were plated at a density of 1 × 10^5 cells/mL. After 12 h, the confluence of the cells reached approximately 40%. The cells were subsequently transduced with lentivirus (MOI = 10) and maintained in DMEM without fetal bovine serum for 8 to 12 h, after which the medium was replaced with complete culture medium. The cells were transduced with lentivirus for a total duration of 72 h.

### Quantitative Real-Time Polymerase Chain Reaction (qRT-PCR)

Mouse lungs tissues was used to extract total RNA by using a high purity RNA extraction kit-RNAiso Plus reagent (TaKaRa, Japan). After quantification, RNA(2 µg) was used to generate first-strand cDNA and then the cDNA were used for qPCR as described previously [6]. The RSV NS1 gene was quantified using TaqMan RT-PCR, as described previously [14].

### Western Blot Analysis

Mice lung homogenates, total cell proteins, cell nuclear and cytoplasmic proteins were extracted using the Nuclear Extraction Kit (Active Motif, USA). Brifly, wash the cells and add 3 mL of ice-cold PBS/Phosphatase inhibitors, collect the cells and transfer them to a pre-chilled 15 mL centrifuge tube. Centrifuge at 200 g for 5 min at 4℃, discard the supernatant, and retain the cell pellet. Add 500 μl of 1X Hypotonic Buffer to resuspend the cell pellet and transfer it to a pre-chilled EP tube, incubating on ice for 15 min to allow the cells to swell. Shake at the maximum speed for 10 s, then centrifuge at 14,000 g for 30 s at 4℃, collecting the supernatant as the cytoplasmic protein.Then Add 50 μl of Complete Lysis Buffer to the cell pellet, thoroughly mix by pipetting and place on ice. Incubate on a shaking platform at 150 rpm for 30 min, shake at maximum speed for 30 s, and centrifuge at 14,000 g for 10 min at 4℃, collecting the supernatant as the nuclear protein. Samples containing equal quantities of protein were separated using 8% or 10% SDS-PAGE and then transferred onto polyvinylidene difluoride (PVDF) membranes. The membranes were then probed with primary antibodies against HMGB1 (1:1 000; Proteintech Group, USA), H1.0(1:1 000; Proteintech Group, USA), NS1 (1:1 000, provide by our labratory), GFP (1:1 000; Proteintech Group, USA), and GAPDH (1:5 000; Proteintech Group, USA). Followed by appropriate alkaline phosphatase-conjugated goat anti-rabbit secondary antibody (1:10 000; Proteintech Group, USA), rabbit anti-goat antibody (1:10 000; Proteintech Group, USA), and goat anti-mouse antibody (1:10 000; Proteintech Group, USA). Signals were quantified using ImageJ 1.52a.

### Immunofluorescence Analysis

The A549 cells were grown on round coverslips, fixed with 4% paraformaldehyde, and blocked using 5% bovine serum albumin as described in our previous work [6]. The coverslips were then incubated with anti-HMGB1 or anti-H1.0 antibodies (1:50; Proteintech Group, USA) overnight at 4 °C, followed by appropriate fluorescein isothiocyanate-conjugated secondary antibody (1:200; Beyotime, China) for 1 h at room temperature. Finally, the cells slips were photographed using a fluorescence microscope (Olympus, Japan) and analyzed with ImageJ 1.52a.

### Immunoprecipitation

A549 cell lysates were used for immunoprecipitation as described in our previous work [5]. Briefly, 80 μL SureBeads Protein G (Bio-Rad, USA) was thoroughly resuspended in 1 ml PBS, then transferred to 1.5-mL tubes. The beads were magnetized and 4 μg of HMGB1, NS1 and H1.0 antibody was added to the beads, followed by rotation for one hour at room temperature. Then, cell lysate (400 μL) was added into the beads and rotated for 2 h at room temperature. Before the final magnetization, the resuspended beads were transferred to a new tube, after which 100 μL of 1 × Laemmli buffer was added and incubated for 10 min at 70 °C.

### Glutathione S-transferase(GST) Pull-Down Assays

GST, GST-NS1, HA-HMGB1 and His-H1.0 fusion proteins were expressed in E. coli BL21 cells. Subsequently, GST-NS1 was purified by using Glutathione Sepharose (GE Healthcare Life Sciences) as described in our previous work [6]; The HA-HMGB1 and His-H1.0 were purified by using Ni–NTA Sepharose. 30 μg of appropriate GST or His fusion proteins coupled to GST or His tag purification resin was mixed at 4 °C for 2 h, then the target fusion protein was added and mixed overnight at 4 °C. The mixed proteins were washed with eluent and dectected by western blotting.

### Surface Plasmon Resonance Analysis

Surface plasmon resonance (SPR)-based measurements were conducted using a BIAcore T200 system (Cytiva). His-NS1 and His-H1.0 fusion proteins were expressed in E. coli BL21 cells and purified. The NS1 protein was diluted to 20 μg/mL in 10 mM acetate buffer at pH 5.5, with a flow rate of 10 μL/min, and immobilized on the CM5 chip(Series Sensor Chip CM5, Cytiva) to a density of 600 RU in target density mode. Before this procedure, the sensor surface was activated by injecting a mixture of 50 mM N-Hydroxysuccinimide and 200 mM 1-Ethyl-3-(3-dimethylaminopropyl)carbodiimide for 7 min. Finally, the surface was blocked with 1 M ethanolamine at pH 8.5. For affinity analysis, the H1.0 protein was dissolved in PBS buffer at concentrations of 0, 15.625, 31.25, 62.5, 125, 250, and 500 nM, and then flowed across the chip. After binding to the surface, each sample was dissociated in PBS buffer for 360 s at a flow rate of 30 μL/min. The dissociation constant (KD) was determined and recorded using the BIAcore T200 system.

### In Silico Study

The structures of Histone H1.0 and NS1 proteins were obtained from the PDB database (7COW and 5VJ2 respectily, which only the globular portion of H1.0 was utilized for docking). To process the protein structures for further analysis, the H +  + 3 online server performed protonation processing under neutral conditions at a pH of 7. Subsequently, UCSF Chimera software was employed to eliminate impurities and water molecules from the crystal structure, leaving only the protein structure with allocation of Amber14SB charges. Protein docking was then performed using the professional tool HDOCK. Molecular docking and conformational scoring were performed using the empirical iterative scoring function ITScorePP, a negative score indicates molecular binding, and a larger absolute value signifies a stronger binding ability.The maximum number of output conformations for docking was set to 100, and the top 10 conformations were scored. A confidence score was employed for reliability analysis, where a score greater than 0.7 indicates that the docking score is reliable and that there is a high likelihood of molecular binding. The conformation with both the highest docking score and confidence score was selected for subsequent analysis.The 3D mapping analysis was conducted using PyMol v2.5.7, while the academic version of Maestro software was utilized for 2D interaction and statistical analysis of interaction characteristics, distances, and quantities. Following this, the protein complex resulting from the docking process underwent a 100 ns molecular dynamics simulation using the GROMACS software (version: 2020, Phad Calculation, Chengdu, China.), with the force field parameter set to AMBER99sb-ildn. Based on the outcomes of the molecular dynamics simulations, analyses were performed on the Root Mean Square Deviation (RMSD), Root Mean Square Fluctuation (RMSF), Radius of Gyration (Rg), Solvent Accessible Surface Area (SASA), and Hydrogen Bonds (H-bonds) of the complex, which were graphically presented using GraphPad Prism. The Gibbs free energy was computed utilizing the “g_sham” and “xpm2txt.py” scripts based on RMSD and Rg values.

### Statistical Analysis

All statistical analyses were performed using Prism GraphPad Software 8.4.0(La Jolla, CA, USA), with results expressed as mean ± standard error of the mean (SEM). Analysis of variance (ANOVA) was used to determine differences between groups. Data lacking normal distribution were evaluated using the nonparametric Kruskal–Wallis test. Differences were considered significant at *P* < 0.05.

## Results

### Knockdown of NS1 Reduced Airway Inflammation in the Late-Stage RSV Infection in Mice

**Fig. 1:**
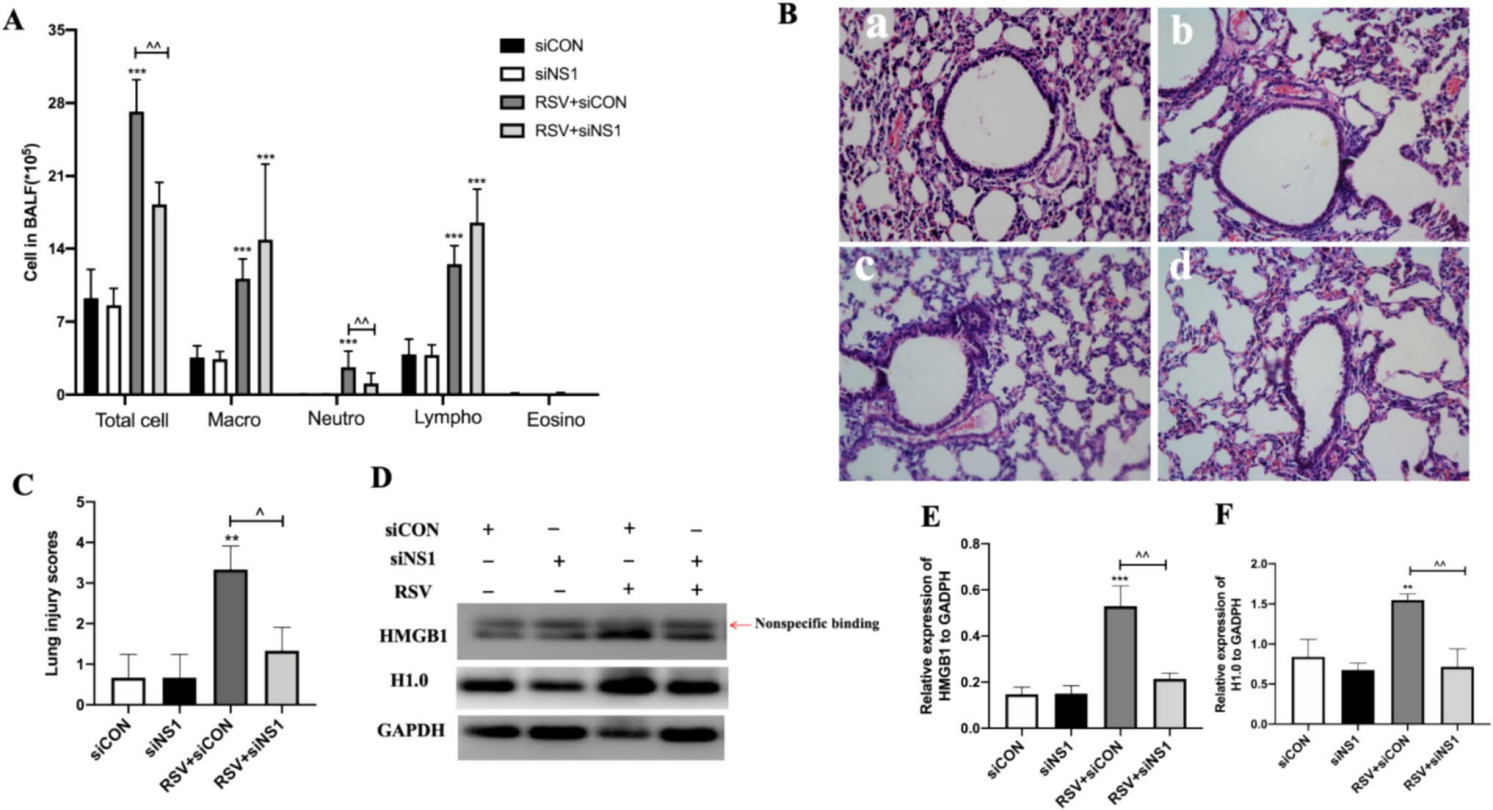
Knockdown of NS1 reduced airway inflammation in late-stage RSV infection in mice. RSV-infected mice, mock mice treated with siCON or siNS1 were assessed for airway inflammation. BALF was collected for inflammatory cell counts. **A** Inflammatory cell number, including total cells, macrophages(Macro), lymphocytes(Lympho), neutrophils(Neutro), and eosinophils(Eosino), in the BALF on day 21 after NS1 knockdown are shown (*n* = 6). **B**-**C** Lung tissue histology was performed on day 21 after RSV infection (a: siCON group; b: siNS1 group; c: RSV + siCON; d: RSV + siNS1. **C** Lung injury scores) (*n* = 3). **D** Expression levels of HMGB1 and H1.0 in lungs. **E**–**F** HMGB1 and H1.0 protein level quantification (*n* = 3). ** and *** represent *P* < 0.001 and *P* < 0.0001, respectively(RSV + siCON or RSV + siNS1 group compared with control siCON group); ^ and ^^ represent *P* < 0.05 and *P* < 0.001 respectively(RSV + siNS1 compared with RSV + siCON group)

Previous research indicates that airway inflammation can persist for up to 60 days following RSV infection in mice, even when RSV replication ceases after day 7 [15, 16]. HMGB1 levels were observed to rise significantly on day 14 and day 21, alongside a notable increase in cytokines, particularly those linked with a type two immune response, on day 21. Treatment with anti-HMGB1 antibodies effectively suppressed airway inflammation and hyperresponsiveness by day 21 [10]. To investigate the impact of the NS1 protein on RSV-induced HMGB1 expression and inflammation, mice received NS1-specific siRNA treatment. This intervention led to a significant decrease in lung airway inflammation. As depicted in Fig. 1, BALF from mice treated with control siRNA (siCON) displayed a high number of various leukocytes, such as neutrophils, lymphocytes, and macrophages, infiltrating on day 21 post-infection (Fig. 1A). Conversely, the use of siNS1 plasmid reduced the total leukocyte count and presence of neutrophils in the BALF of RSV-infected mice, correlating with diminished lung tissue damage and histological scores (Fig. 1B-C). The expression of N, NS1 genes and NS1 protein was notably reduced on day 5 following NS1 knockdown (Supplementary Fig. S1A-C).The results indicated that NS1 silencing might affect viral load and alleviate pulmonary inflammation.

To delve deeper into the influence of NS1 on HMGB1 expression, we quantified the levels of HMGB1 and H1.0 proteins in the lungs of RSV-infected mice. Our findings revealed elevated levels of both HMGB1 and H1.0 proteins in the lungs of mice receiving siCON treatment, while a reduction in protein levels was observed in mice treated with siNS1 (Fig. 1D-F). These outcomes suggest that NS1 promotes the expression of HMGB1, potentially mitigate lung inflammation.

### RSV Infection Increased Expression and Release of HMGB1 in A549 Cells

**Fig. 2:**
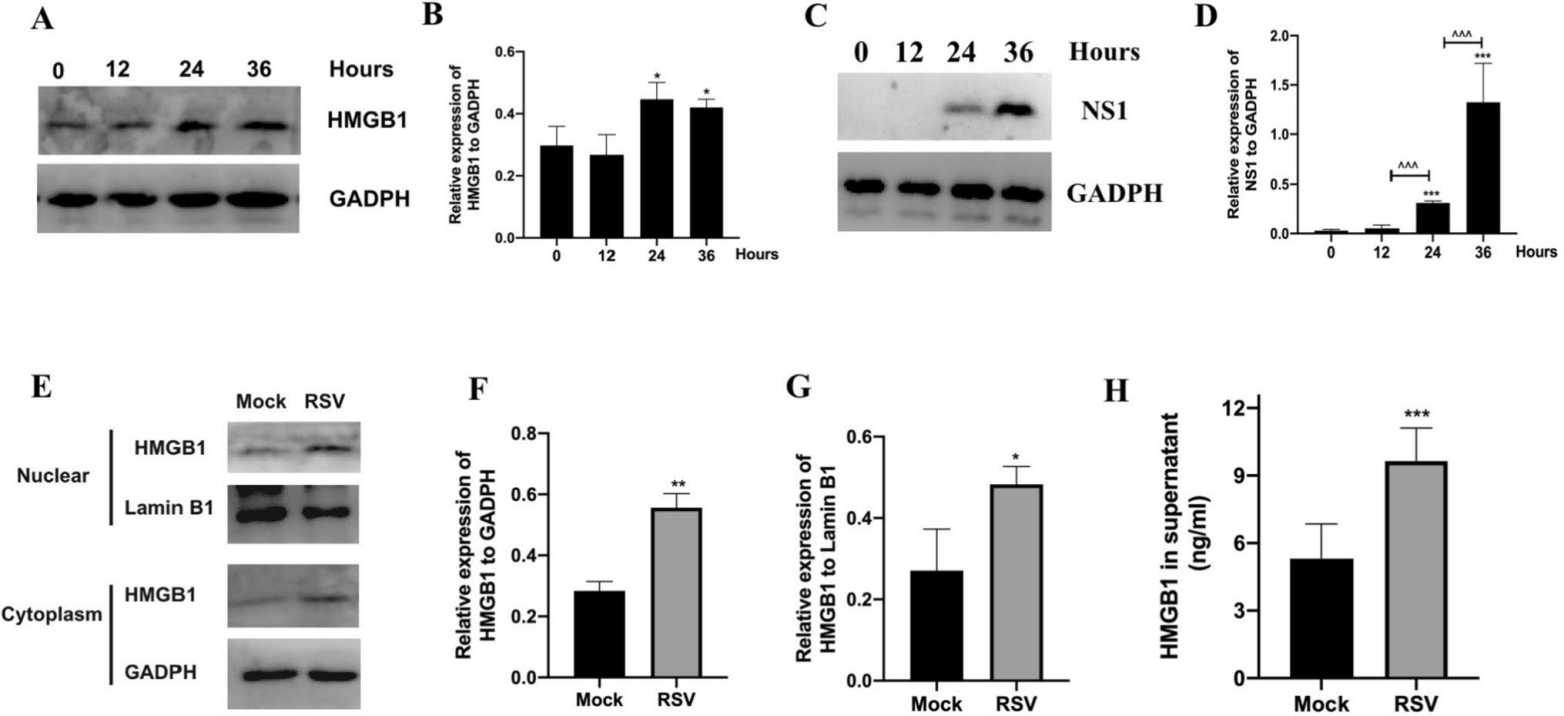
RSV infection increased expression and release of HMGB1 in A549 cells. RSV-infected cells were collected 0, 12, 24, and 36 h after RSV infection. Total protein was extracted for protein expression level assessment. Supernatants were collected at 24 h for HMGB1 detection. HMGB1 (**A**) and NS1 (**C**) protein levels in A549 cells 0, 12, 24, and 36 h after RSV infection (*n* = 3). **B**, **D** Quantification of HMGB1 and NS1 protein levels (*n* = 3). **E** HMGB1 protein levels in cytoplasm and nucleus 24 h after RSV infection. **F**-**G** Quantification of HMGB1 protein levels in cytoplasm and nucleus (*n* = 3). **H** HMGB1 levels in supernatants were measured by ELISA (*n* = 6). *, **, *** represent *P* < 0.05, *P* < 0.01, and *P* < 0.001 respectively(compared with Mock (or 0 h) group). ^^^ represents *P* < 0.001(compared with 24 h group). The Kruskal–Wallis test was employed to compare RSV with the mock group

In RSV-infected A549 cells, the expression levels of HMGB1 and NS1 proteins significantly increased at 24 and 36 h post-RSV infection (Fig. 2A-D). Specifically, HMGB1 expression rose in both the nucleus and cytoplasm at the 24-h mark following RSV infection (Fig. 2E-G), with a concurrent increase in HMGB1 levels in the supernatants after 24 h (Fig. 2H). This indicates that RSV infection stimulated both the expression and release of HMGB1 in A549 cells.

### Knockdown of NS1 Reduced HMGB1 and H1.0 Expression and Release in A549 Cells

**Fig. 3:**
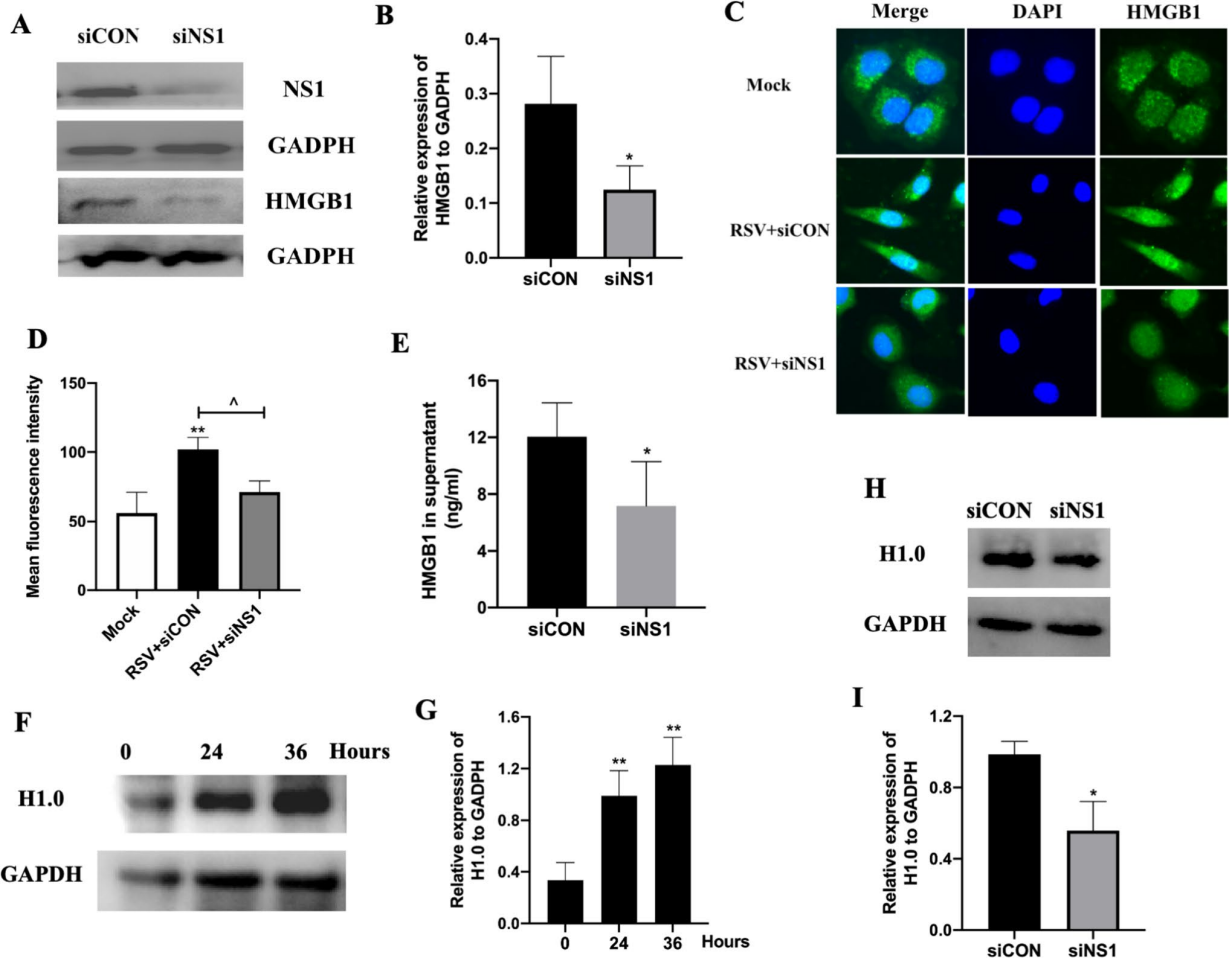
Knockdown of NS1 reduced HMGB1 and H1.0 expression and release in A549 cells. A549 cells were infected with RSV for 6 h, after which siCON and siNS1 were transinfected for another 24 h. **A** HMGB1 protein levels after NS1 knockdown. **B** Quantification of HMGB1 protein levels (*n* = 3). **C** HMGB1 expression levels within the cytoplasm and nucleus were determined using a fluorescence microscope. **D** Mean fluorescence was determined by Image-Pro Plus (*n* = 3). **E** HMGB1 levels in the supernatants, with means ± standard deviation (SD) shown for six independent experiments (*n* = 6). **F** H1.0 protein levels 24 and 36 h after RSV infection. **G** Quantification of H1.0 protein levels (*n* = 3). **H**-**I** Quantification of H1.0 protein levels after NS1 knockdown (*n* = 3). * and ** represent *P* < 0.05 and *P* < 0.01, respectively(siNS1 compared with siCON group or compared with 0 h in panel G). ^ RSV + siNS1 group compared with RSV + siCON (*P* < 0.05). The Kruskal–Wallis test was employed to compare siNS1 with the siCON group

RSV infection triggers the upregulation of HMGB1, whereas treatment with siNS1 leads to a decrease in HMGB1 expression in RSV-infected A549 cells compared to those treated with control siRNA (Fig. 3A-B). Immunofluorescence analysis showed an elevation in HMGB1 expression post-RSV infection in siCON-treated cells, while HMGB1 levels declined with siNS1 treatment (Fig. 3C-D). Additionally, ELISA analysis of the supernatant illustrated that siNS1 treatment hampers the release of HMGB1 post-RSV infection compared to siCON treatment (Fig. 3E). Previous studies have suggested that H1.0 can displace HMGB1 from nucleosomal DNA, leading to HMGB1 release [17]. In this investigation, an escalation in H1.0 expression was observed 24 h post-RSV infection (Fig. 3F-G), with H1.0 levels decreasing after siNS1 treatment compared to siCON treatment (Fig. 3H-I), along with the N gene (Supplementary Fig. S1D). These observations indicate that NS1 knockdown diminishes the production of HMGB1 and H1.0, as well as the release of HMGB1 into the extracellular milieu.

## Knockdown of NS1 Reduced HMGB1 and H1.0 Expression in 16HBE Cells

**Fig. 4:**
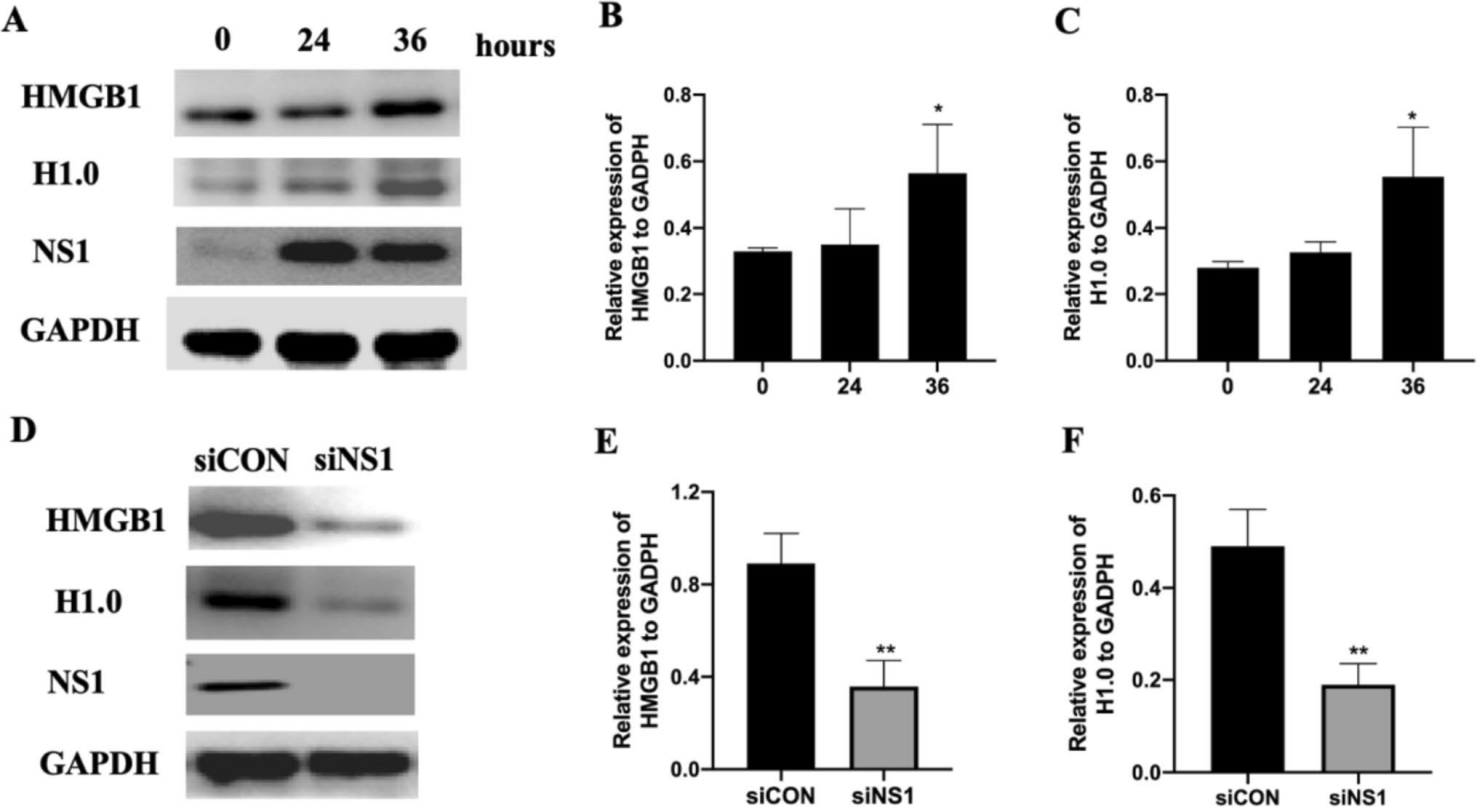
Knockdown of NS1 reduced HMGB1 and H1.0 expression in 16HBE cells. RSV-infected 16HBE cells were collected 0, 24, and 36 h after RSV infection. Total protein was extracted for protein expression level assessment. For siNS1 knockdown, 16HBE cells were infected with RSV for 6 h, then siCON or siNS1 was transinfected for another 24 h. **A** HMGB1 and H1.0 protein levels in 16HBE cells 0, 24, and 36 h after RSV infection (*n* = 3). **B**-**C** Quantification of HMGB1 and H1.0 protein levels (*n* = 3). **D** HMGB1 and H1.0 protein levels after NS1 knockdown. **E**–**F** Quantification of HMGB1 and H1.0 protein levels (*n* = 3). * represent *P* < 0.05(compared with 0 h represent); ** represent *P* < 0.01(compared with siCON group). The Kruskal–Wallis test was employed to compare siNS1 with the siCON group

We subsequently employed 16HBE cells to validate the essential role of NS1 in HMGB1 expression. The levels of HMGB1 and H1.0 proteins exhibited a noticeable increase at 36 h post RSV infection (Fig. 4A-C). Nevertheless, siNS1 treatment resulted in a reduction in the protein expression levels of both HMGB1 and H1.0 (Fig. 4D-F).

### Overexpression of NS1 Increased HMGB1 Expression in A549 Cells

**Fig. 5:**
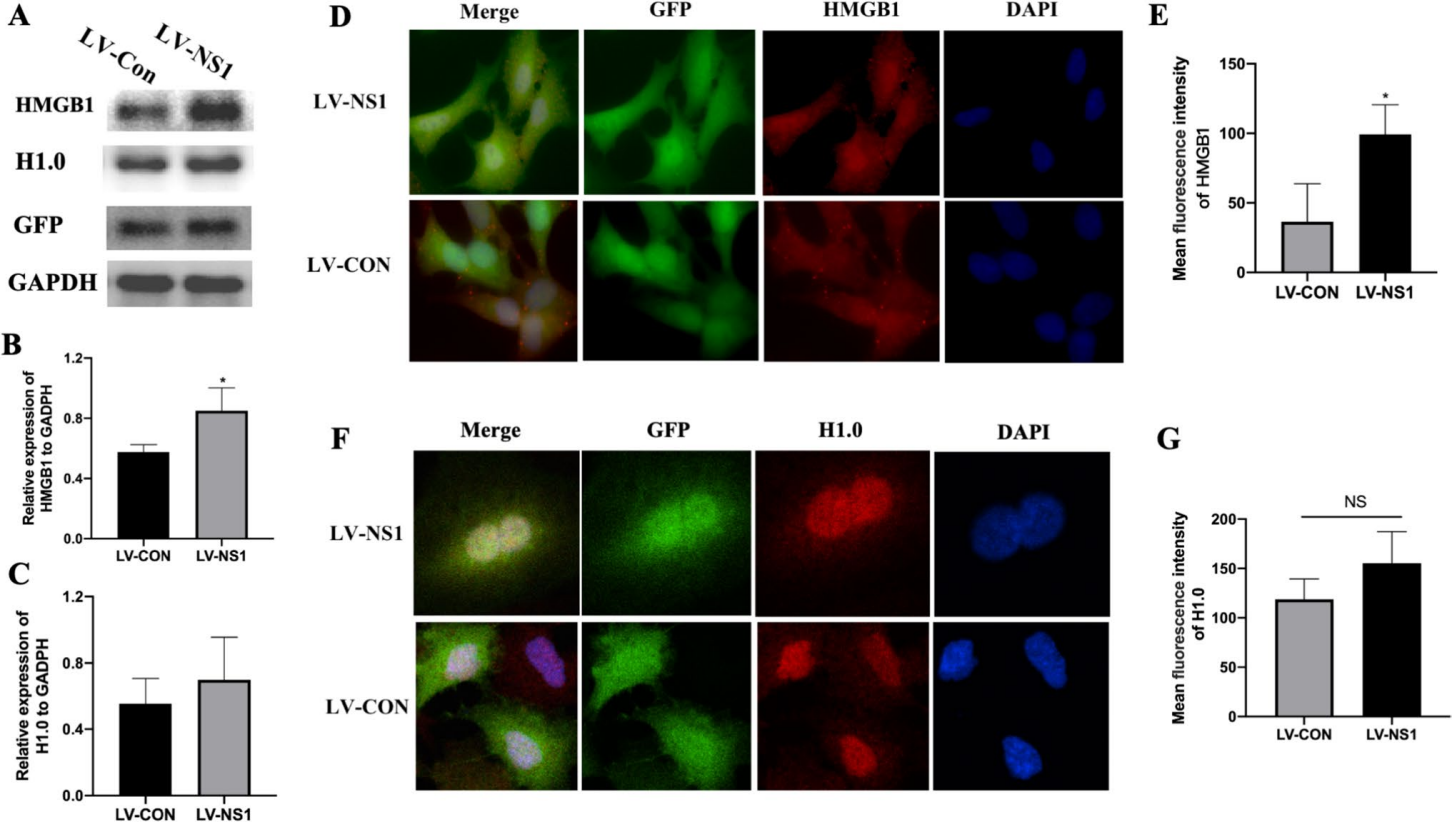
Overexpression of NS1 increased HMGB1 expression in A549 cells. A549 cells were treated with NS1 or control lentivirus for 72 h, then collected for protein detection or immunofluorescence analysis. **A**-**C** HMGB1 and H1.0 protein levels after overexpression of NS1 (**A**, the GFP inbdicated lentivirus transfection) and quantification of HMGB1 (**B**) and H1.0 (**C**) protein levels (*n* = 3). **D** HMGB1 expression within the cytoplasm and nucleus determined by fluorescence microscope. **E** Mean fluorescence of HMGB1 determined by Image-Pro Plus (*n* = 3). **F** H1.0 expression within the nucleus determined by fluorescence microscope. **G** Mean fluorescence of H1.0 determined by Image-Pro Plus (*n* = 3). * represent *P* < 0.05(siNS1 compared with siCON group).The Kruskal–Wallis test was employed to compare LV-NS1 with the LV-CON group

Following the reduction in HMGB1 and H1.0 expression due to NS1 knockdown, we proceeded to assess the direct influence of NS1 on HMGB1 production in A549 cells. The cells were exposed to NS1 or control lentivirus for 72 h, after which protein collection was conducted for analysis. In NS1 lentivirus-infected cells, HMGB1 expression increased, while H1.0 expression remained constant (Fig. 5A-C). Moreover, immunofluorescence analysis revealed an upregulation of HMGB1 by NS1 (Fig. 5D-E) with no impact on H1.0 levels (Fig. 5F-G). Furthermore, NS1 was mainly localized in the nucleus with colocalization observed with H1.0 (Fig. 5F) but not with HMGB1.

### NS1 Directly Bound to H1.0 and Inhibited Binding of HMGB1 to H1.0

**Fig. 6:**
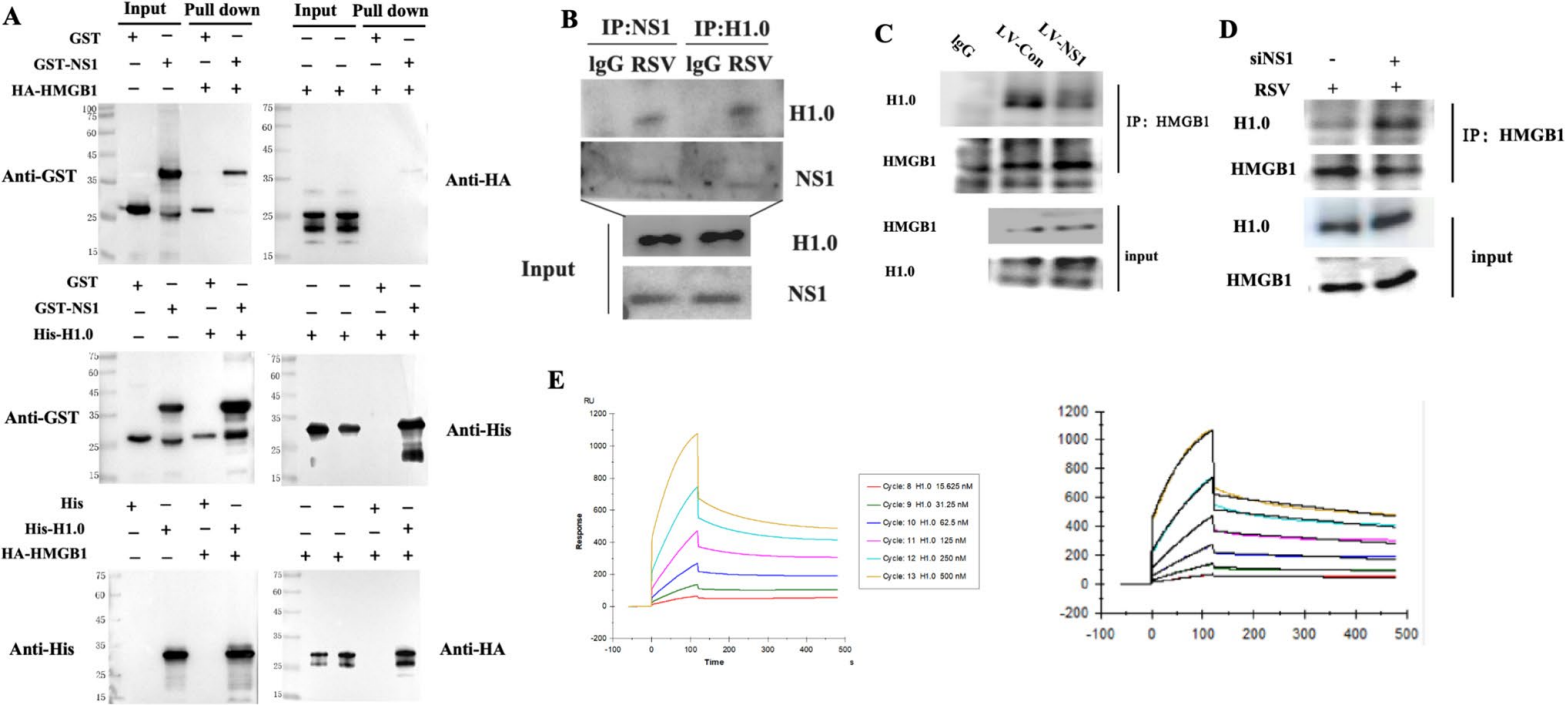
NS1 directly bound to H1.0 and inhibited binding of HMGB1 to H1.0. **A** GST-pulldown was used to detect direct binding of NS1, HMGB1, and H1.0. **B** Immunoprecipitation was used to detect direct binding of NS1 to H1.0 in A549 cells after RSV infection for 24 h. **C**-**D** Immunoprecipitation was used to detect direct binding of HMGB1 to H1.0 in A549 cells after overexpression of NS1 for 72 h (C) and after RSV infection for 6 h and siNS1 treatment for 24 h (D). **E** Surface plasmon resonance analysis revealed that the dissociation constant (KD) for the binding of NS1 to H1.0 was measured at 31.19 nM; the association rate constant(ka) was 2.536 × 10^4^(1/Ms);The bulk effect could be coused by the glycerol in the buffer of H1.0 stock solution. Experiment was repeated at least two times

To further investigate the interactions between NS1 and H1.0, we conducted GST pull-down and immunoprecipitation assays to detect direct binding. The results indicated that NS1 directly binds to H1.0, but not to HMGB1, whereas HMGB1 directly binds to H1.0 (Fig. 6A-B). Additionally, cells infected with NS1 lentivirus showed an decreased binding of H1.0 to HMGB1 compared to cells infected with the control lentivirus (Fig. 6C). Conversely, RSV-infected cells treated with siNS1 exhibited a increase binding of H1.0 to HMGB1 compared to cells treated with siCON (Fig. 6D). Furthermore, surface plasmon resonance analysis showed that the dissociation constant (KD) for the binding of NS1 to H1.0 was measured at 31.19 nM and the association rate constant(ka) was 2.536 × 10^4^(1/Ms) (Fig. 6E).

**Fig. 7:**
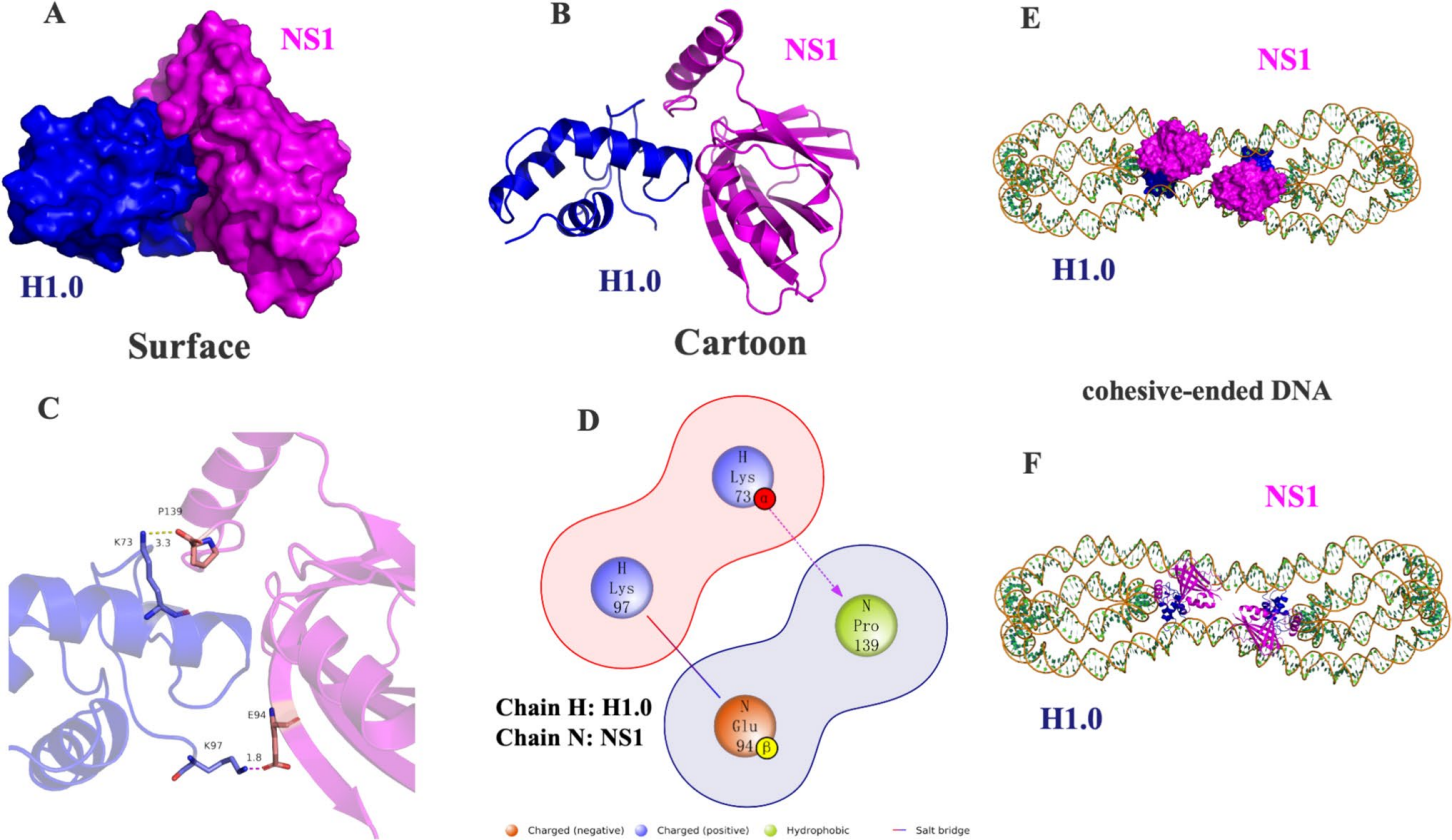
Molecular interactions between NS1 and H1.0 in silico. The 3D protein complex structure, in surface (**A**) and in cartoon (**B**) representation, which H1.0 protein in blue, NS1 protein in Magenta, and the interaction is represented by dotted lines. **C**-**D** The 2D protein complex structure shows blue amino acids representing basic amino acids, green indicating hydrophobic amino acids, and red denoting acidic amino acids. Salt bridges and hydrogen bond interactions are represented using solid lines and dashed lines, respectively. **E**–**F** 3D structure models of a NS1-H1.0-DNA ternary complex. The DNA double stranded backbone is displayed in orange, and the base is displayed in green

An in silico study uncovered extensive binding between NS1 and H1.0 proteins, involving notable interactions such as hydrogen bonds and salt bridges (Fig. 7A-D). This model exhibited the highest docking score of −328.57 kcal/mol, accompanied by a confidence score of 0.9727, which indicates a high level of reliability for the results.

**Fig. 8:**
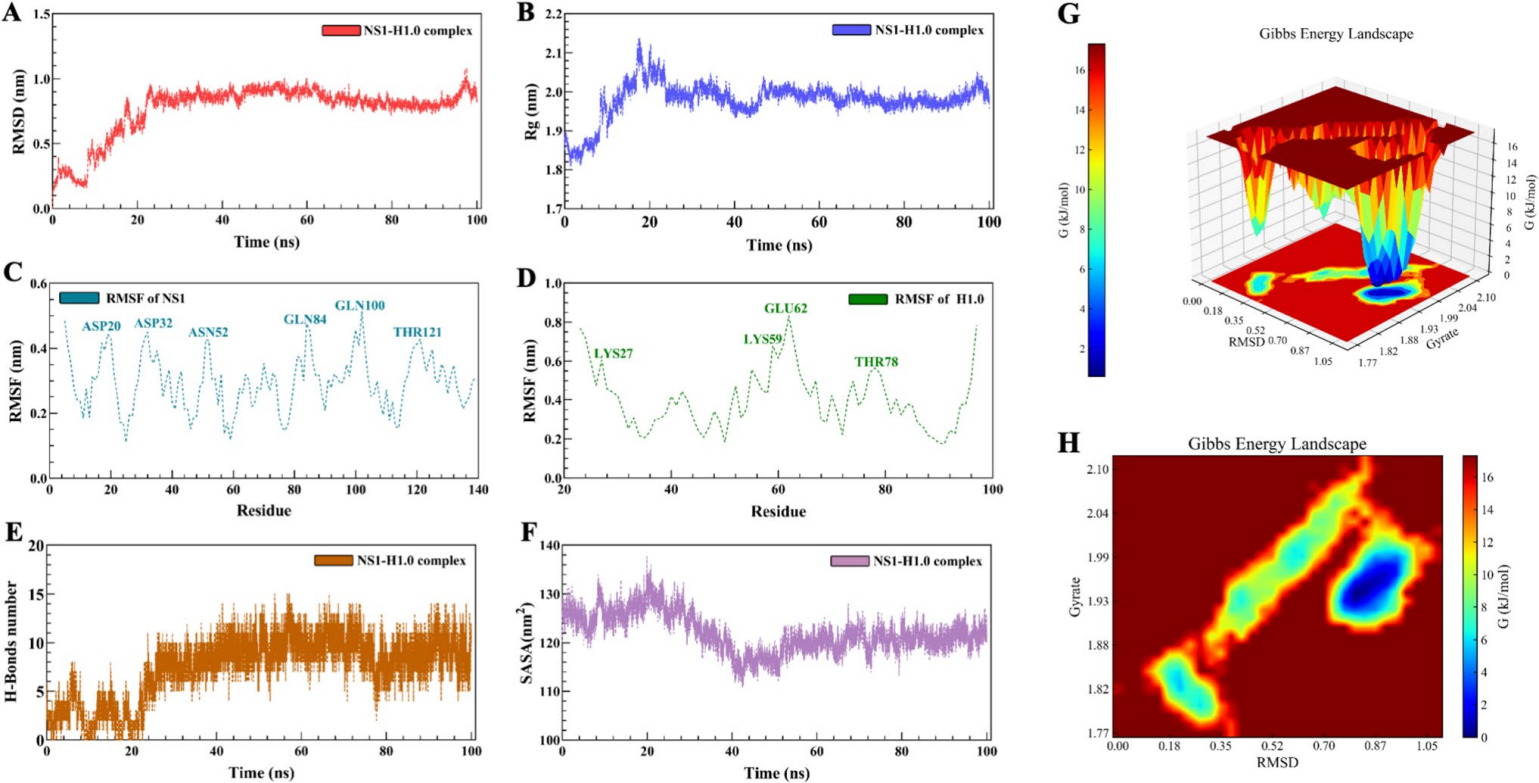
The stability of the complex through kinetic analysis. **A** The root mean square deviation (RMSD) curve of the complex; **B** The radius of gyration (Rg) curve; **C** Root Mean Square Fluctuation (RMSF) values of NS1; **D** Root Mean Square Fluctuation (RMSF) values of H1.0; **E** The number of hydrogen bonds between the complexes; **F** The curve of solution accessible surface area (SASA); **G** Gibbs free energy 3D morphology maps; **H** Gibbs free energy 2D morphology maps

Notably, Pro139 on NS1 and Lys73 on H1.0 formed an interaction, with Lys73, an alkaline and positively charged amino acid, establishing hydrogen bonds with Pro139 at a distance of 3.3 Å. Another interaction involved Glu94 on NS1 and Lys97 on H1.0, forming a salt bridge at a distance of 1.8 Å (Fig. 7C-D). Furthermore, we assessed the stability of the complex through kinetic analysis. The root mean square deviation (RMSD) curve of the complex reached equilibrium after an initial rise, stabilizing at 0.8 nm RMSD after 24 ns (Fig. 8A). Similarly, the radius of gyration (Rg) curve exhibited an overall decreasing trend, stabilizing around 2 nm after 48–96 ns (Fig. 8B). Analysis of the NS1 structure indicated significant Root Mean Square Fluctuation (RMSF) values for ASP20, ASP32, ASN52, GLN84, GLN100 and THR121 residues, while the H1.0 protein structure showed notable flexibility in residues LYS27, LYS59, GLU62 and THR78 during the simulation (Fig. 8C-D). Moreover, an increase in the number of hydrogen bonds between the complexes was observed over the simulation time, promoting interaction (Fig. 8E). The solution accessible surface area (SASA) of the complexs decreased for a period of time and stabilized after 52 ns, with a mean SASA of 120 nm^2^ (Fig. 8F). Additionally, Gibbs free energy calculations based on RMSD and Rg values portrayed distinct lowest energy regions in the 3D and 2D morphology maps (Fig. 8G-H), signifying strong and stable interactions within the composite structure.These findings provide valuable insights into the specific interactions that contribute to the binding between NS1 and H1.0 proteins. However, this model cannot fully account for a direct competition between NS1 and HMGB1 for H1.0 binding, since NS1 binds to the globular domain of H1.0, while HMGB1 binds to the C-terminal tail of H1.0.

## Discussion

The results of our study underscore the significant role of NS1 in promoting airway inflammation during late-stage RSV infection. Notably, NS1 was found to induce the expression and release of HMGB1 in airway epithelial cells by directly binding to H1.0. Furthermore, when the NS1 gene was silenced, it led to a reduction in inflammation mediated by RSV infection partially through the inhibition of HMGB1 production. To the best of our knowledge, our study is the first to demonstrate that NS1 (i) directly interacts with H1.0 in airway epithelial cells, (ii) inhibits the binding of HMGB1 to H1.0, (iii) induces the expression and release of HMGB1, and (iv) knockdown of NS1 mitigates airway inflammation in mice during the late-stage of RSV infection. These findings suggest that NS1 has the potential to be targeted for therapeutic interventions following RSV infection.

HMGB1 serves as a structural component of chromatin within the nucleus, contributing to the maintenance of chromosome structure and stability. It consists of two DNA-binding domains and an acidic carboxyl terminus that can interact with histones H1 and H3, and can also form dimers with H1 [18, 19]. Upon encountering extracellular stimuli, HMGB1 initially dissociates from the nucleus and translocates to the cytoplasm, before ultimately being released into the extracellular space through a series of intricate processes, hence participating in inflammatory responses [20, 21]. In our previous study, we elucidated the significant role of HMGB1 in promoting inflammation in mice during the late-stage of RSV infection [10]. In this study, we demonstrate that knocking down NS1 led to reduced HMGB1 protein levels and suppression of airway inflammation in late-stage RSV infection. Additionally, knockdown of NS1 in A549 and 16HBE cells resulted in decreased protein levels of HMGB1 and its extracellular release. Therefore, our findings suggest that NS1 facilitates the expression and secretion of HMGB1.

NS1 interacts with multiple host proteins in the nucleus, regulating host gene transcription by interacting with host nuclear components [22]. A previous study has demonstrated that NS1 binds directly to histone H2BD and enhances HOX gene expression following RSV infection [13]. Thus, NS1 potentially interacts with histones and impacts HMGB1 expression. The release of HMGB1 from the nucleus is closely linked to the nucleosome. Post-translational modifications of histones H1-H4 can induce chromatin relaxation, consequently facilitating gene expression and the binding of transcription factors to DNA [23, 24]. HMGB1 interacts with H1, influencing the condensation state of chromatin [25], and competes with H1 for binding sites on chromatin [26]. Knocking down HMGB1 does not impact H1 binding to the TNF-α promoter in silenced cells; however, knockdown of H1 substantially reduces HMGB1 binding [17]. Recent research has revealed that adenovirus histone-like protein VII specifically targets the antagonistic relationship between H1 and HMGB1, resulting in the obstruction of the cell cycle [27]. In this study, we demonstrated the direct interaction of both NS1 and HMGB1 with H1.0(variant of H1, which is independent of replication and can be observed in non-replicating cells). The overexpression of NS1 inhibited the interaction between HMGB1 and H1.0, leading to elevated HMGB1 expression. Conversely, the depletion of NS1 enhanced the binding of HMGB1 to H1.0. These results indicate that NS1 interacts with H1.0, thereby promoting HMGB1 expression, impeding the binding of HMGB1 to H1.0, and facilitating its translocation from the nucleus to the cytoplasm and extracellular space.

In conclusion, mounting evidence suggests that NS1 plays a critical role in the upregulation of HMGB1 expression and its release following RSV infection. Both HMGB1 and H1.0 expressions are increased post RSV infection. Moreover, the predominant localization of NS1 in the nucleus, where it binds to H1.0, facilitates the detachment of HMGB1 from H1.0, leading to its subsequent release into the extracellular space, which partially triggers inflammation. The involvement of NS1 in the context of RSV infection becomes more complex as knockdown of NS1 also leads to reduced H1.0 expression. Therefore, further investigation is required to elucidate the precise mechanism by which NS1 induces HMGB1 expression. Moreover, the binding of NS1 to H1.0 may potentially modulate the expression of specific genes and further work is needed to investigate the impact of NS1 binding to H1.0. This study advances our understanding of the functional role of NS1.

## Supplementary Information

Below is the link to the electronic supplementary material.

Supplementary file1 (DOCX 2664 KB)

## Data Availability

No datasets were generated or analysed during the current study.
