# Supplementary material for: Nonstructural Protein 1 Mediates HMGB1 Release by Targeting Histone H1.0 After Respiratory Syncytial Virus Infection In Vivo and In Vitro
Source: Inflammation. 2025 May 14;48(5):3579–91. doi: 10.1007/s10753-025-02285-6 (PMC12596378; doi:10.1007/s10753-025-02285-6)

**Supplementary Figure S1** Expression of NS1, N gene treatment with siNS1 in mice after RSV infection or in A549 cells.

Mouse lungs were collected to quantify NS1(A) , N(B)gene expression using qRT-PCR, with means ± SD shown for three independent experiments (n = 3) . C: NS1 protein expression in mice lung on day 5 after RSV infection. D: quantify N gene expression in A549 cells(n = 6).

**represent *P* < 0.001(siNS1 compared with siCON group ).


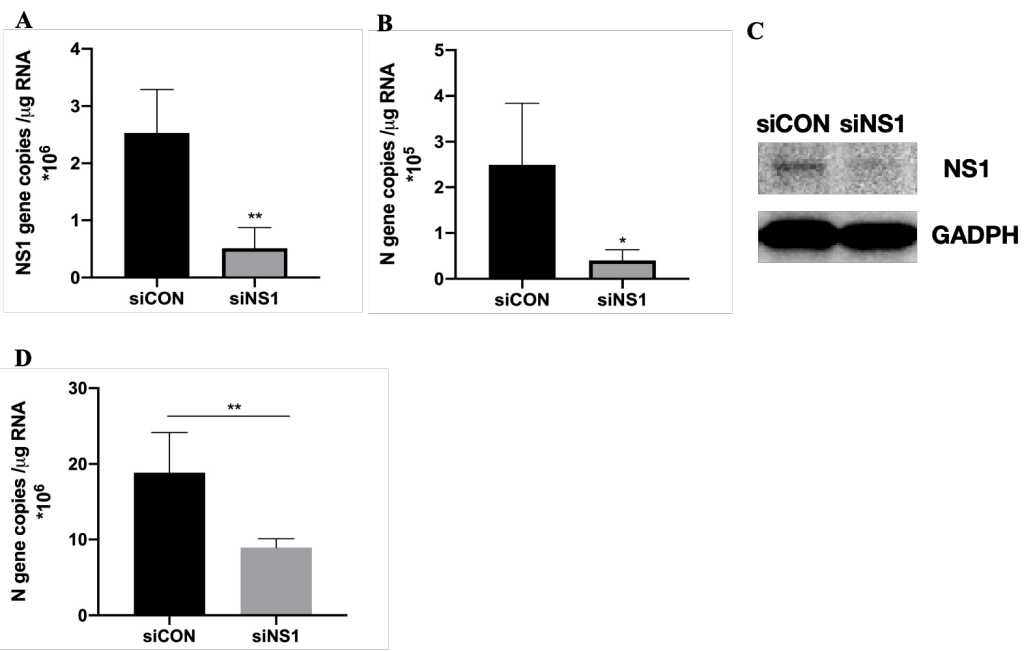

Supplement: Supplementary file 1 — Supplementary file1 (DOCX 2664 KB) [file 10753_2025_2285_MOESM1_ESM.docx]
